# Bilateral Serratus Plane Block in a Critically Ill, Mechanically Ventilated Patient with Multiple Rib Fractures Due to Severe Thoracic Trauma: Case Report and Literature Review

**DOI:** 10.3390/jcm14061864

**Published:** 2025-03-10

**Authors:** Francesco Baccoli, Beatrice Brunoni, Francesco Zadek, Alessandra Papoff, Lorenzo Paveri, Vito Torrano, Roberto Fumagalli, Thomas Langer

**Affiliations:** 1 Department of Medicine and Surgery, University of Milan-Bicocca, 20900 Monza, Italy; f.baccoli@campus.unimib.it (F.B.); b.brunoni@campus.unimib.it (B.B.); francesco.zadek@unimib.it (F.Z.); roberto.fumagalli@unimib.it (R.F.); 2 Department of Anesthesia and Intensive Care Medicina, Niguarda Ca’ Granda, 20162 Milan, Italy; alessandra.papoff@ospedaleniguarda.it (A.P.); vito.torrano@ospedaleniguarda.it (V.T.); 3 Department of Radiology, Niguarda Ca’ Granda, 20162 Milan, Italy; lorenzo.paveri@ospedaleniguarda.it

**Keywords:** critical care, regional anesthesia, pain management, local anesthetics, rib fractures, polytrauma, mechanical ventilator weaning

## Abstract

**Background/Objectives**: Effective pain management in polytrauma patients with rib fractures is essential, particularly in the critical care setting. While epidural analgesia is considered the gold standard, it is not always feasible, necessitating alternative locoregional approaches. We present the case of a polytrauma patient with multiple, bilateral rib fractures and severe chest pain that hindered weaning from mechanical ventilation. A bilateral Serratus Anterior Plane Block (SAPB) was performed, with catheters placed for continuous administration of local anesthetics. Pain relief was immediate, enabling a rapid weaning from mechanical ventilation, safe extubation, and subsequent discharge to rehabilitation. A review of the literature on this technique in critically ill patients with thoracic trauma and multiple rib fractures is also presented. **Methods**: We conducted a literature search up to November 2024, identifying studies evaluating the use of SAPB in critically ill patients with chest trauma and rib fractures. **Results:** Eight studies were identified, including a total of 197 cases, of which only 3 involved a bilateral SAPB. Studies and published case reports demonstrated significant variability in analgesic protocols and reported outcomes. Notably, only two papers addressed specifically its role in facilitating weaning from mechanical ventilation. **Conclusions**: Pain control is fundamental in managing severe chest trauma. This case and the reviewed literature suggest that the SAPB is a promising option when epidural analgesia is contraindicated or impractical. However, further studies are needed to define its place in clinical practice and optimize its use in critically ill patients.

## 1. Introduction

Trauma is a leading cause of death, especially among young adults, affecting their prime years of productivity [1]. Additionally, survivors of trauma often experience significant and permanent disabilities. Chest trauma is common in polytrauma patients, occurring in approximately 65% of cases [2]. Moreover, it contributes significantly to mortality, as thoracic injuries are an independent predictor of 30-day mortality [3,4]. Rib fractures are frequent findings in this context and are typically associated with significant chest pain. The incidence of rib fractures increases, especially among elderly patients [4]. While treating chest trauma is certainly challenging, managing pain is considered a medical emergency [5], as uncontrolled pain leads to impaired cough, high sedation requirements, and longer mechanical ventilation support. This, in turn, increases the risks of developing pneumonia and other infections, leading to prolonged hospital and Intensive Care Unit (ICU) length of stay and, ultimately, increased mortality [6].

Epidural analgesia has traditionally been regarded as the cornerstone of pain management for multiple rib fractures [6,7]. However, several patients’ conditions could limit the feasibility of this technique [8]. In addition, conflicting evidence exists regarding its impact on clinical outcomes [6,7]. As a result, alternative regional anesthesia techniques have been explored for managing chest trauma.

The Serratus Anterior Plane Block (SAPB) was first introduced for the management of chest pain following breast surgery [9]. This block targets the lateral cutaneous branches of the intercostal nerves. It offers two ultrasound-guided approaches: the “superficial serratus anterior plane block”, which is performed by injecting local anesthetic between the *Serratus* and *Latissimus Dorsi* muscles, and the “Deep Serratus Anterior Plane Block” technique, obtained by placing the needle below the *Serratus* muscle. This technique presents a straightforward and secure approach of anesthetizing the lateral hemithorax [9]. Its potential application in various contexts, including thoracic trauma, has prompted several authors to explore its efficacy [10]. Notably, the SABP also seems to be effective in posterior rib fractures [11]. A cadaveric study might explain this finding by indicating anatomical post-injury alterations that facilitate the spread of local anesthetics into deeper planes [12,13].

Despite these promising results, this technique is still underused in ICUs [14].

In this paper, we present a case report about the successful use of a continuous, bilateral SAPB as a safe and effective analgesic strategy to treat severe chest pain following bilateral and multiple rib fractures in a patient admitted to our ICU. Furthermore, we reviewed the English literature on this topic to assess the role of this block in the critical care setting.

## 2. Materials and Methods

This case report adheres to the applicable Enhancing the QUAlity and Transparency Of Health Research (EQUATOR) guidelines. For the literature review, we conducted a PubMed search using the following Medical Subject Headings (MeSH) terms: “Nerve Block” [MAJR] or “Rib Fractures/complications” [MAJR] or “Rib Fractures/drug therapy” [MAJR] or “Pain Management/methods” [MAJR] or “Rib Fractures/therapy” [MAJR] or “Nerve Block/methods”[MAJR] and an Embase search according to the equivalent Emtree terms: (((‘nerve block’/exp OR ‘rib fracture’/exp) AND (‘complication’/exp OR ‘drug therapy’/exp OR ‘therapy’/exp) OR ‘pain management’/exp) AND ‘method’/exp OR ‘nerve block’/exp) AND ‘method’/exp AND [english]/lim AND [1 November 2024]/sd. Studies published in English up to November 2024 were considered, including relevant references cited in eligible articles. Titles and abstracts were screened to identify papers describing the use of Serratus Anterior Plane Block (SAPB) or comparing different regional anesthesia techniques. Both original research and review articles were manually reviewed. Only studies involving critically ill patients treated in the Intensive Care Unit (ICU) were included.

From each selected manuscript, we extracted the following data: title, first author, year of publication, study design, number of patients, unilateral or bilateral block, rib involvement (number and description), single-shot injection or continuous infusion, type and concentration of local anesthetic or adjuvants, catheter management (if applicable), and a summary of the results.

## 3. Results

### 3.1. Case Presentation

**Figure 1:**
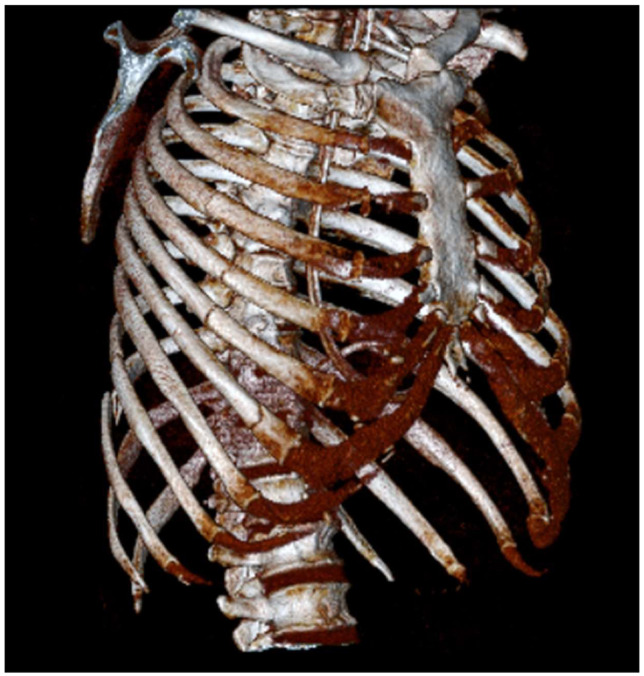
Tridimensional reconstruction of the chest wall, view of the right hemithorax with rib fractures extending from the third to the twelfth.

A 59-year-old man, injured in a motor vehicle crash, was admitted to the trauma center of the Niguarda Hospital in Milan. He had a medical history of arterial hypertension. His initial Glasgow Coma Scale (GCS) score was 14, which rapidly deteriorated to 11, prompting immediate intubation. Due to persistent hypotension despite fluid boluses, a left thoracostomy was performed on suspicion of a tension pneumothorax. Upon arrival in the emergency department, the protocol for massive transfusion was activated, and norepinephrine was administered due to persisting hemodynamic instability. The main Computed Tomography findings were a subarachnoid hemorrhage without surgical indication, an unstable pelvic fracture, and bilateral rib fractures extending from the third to the twelfth on the right side and from the third to the sixth on the left side (Figure 1). There were no signs of active bleeding. Urgent stabilization of the pelvic fracture was performed, following which the patient, now hemodynamically stable, was transferred to our ICU.

**Figure 2:**
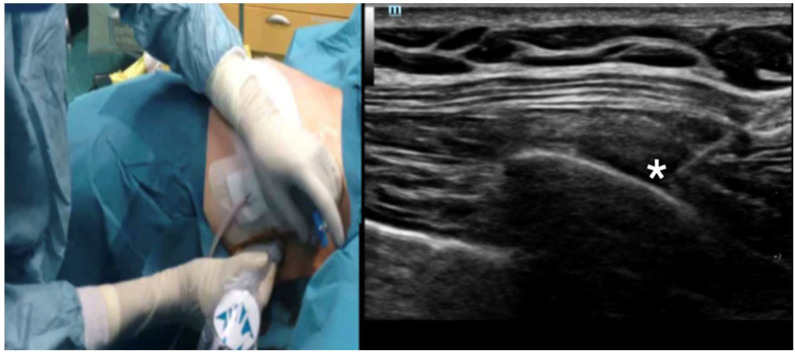
Technique for positioning of the catheter for continuous analgesia in Serratus Anterior Plane Block. Left panel: catheter placement via a sterile technique. Right panel: ultrasound in-plane view of the maneuver. The needle tip was positioned between the Serratus muscle and the surface of the fifth rib in a craniocaudal direction, using the “deep technique”. The * marks the needle tip in the fascial plane between the rib and the Serratus muscle.

Due to severe bilateral chest pain, the placement of a thoracic epidural catheter was attempted, without success. As a consequence, the patient required high doses of intravenous opioids (remifentanil 0.12 µg/kg/min) and sedatives (propofol 3 mg/Kg/h) to guarantee adequate pain control. This, in turn, hindered the possibility of ventilatory weaning and extubation despite the absence of significant lung parenchyma involvement. Four days after the trauma, we attempted an ultrasound-guided continuous bilateral serratus plane block using a linear ultrasound probe and an 18 gauge/50 mm needle (Silverstim 50 mm-30°; Vygon, Ecouen, France). The procedure was performed in-plane, following a catheter-over-needle approach [15,16]. The tip was positioned between the Serratus muscle and the surface of the fifth rib in a craniocaudal direction, using the “deep technique” (Figure 2). After hydro-dissection, we advanced the catheter for 8 cm and injected 20 mL of 0.375% ropivacaine bilaterally. Our analgesic plan included the administration of a 20 mL bolus of 0.2% ropivacaine, every 8 h on each side, complemented by acetaminophen (1 g × 4 time/die) and as part of a multimodal analgesic strategy. The patient was closely monitored for cardiovascular and neurological symptoms attributable to local anesthetic toxicity. The benefits of this approach were immediate: opioids and sedatives could be rapidly suspended, enabling successful weaning from mechanical ventilation and subsequent extubation the following day. Thereafter, the patient underwent a second definitive pelvic surgical procedure and was discharged on day 11 to rehabilitation. Notably, no signs or symptoms of local anesthetic systemic toxicity were observed.

### 3.2. Literature Review

Our research string identified 37,846 papers up to November 2024. From these, 35 studies were selected for reporting data from patients with traumatic rib fractures managed with a serratus plane block. Subsequently, we selected only the manuscripts dealing with critically ill patients admitted to the ICU (Figure S1 of the Supplementary Material), yielding the final eight included studies (Table 1). The included studies included three randomized control trials: one comparing SAPB with thoracic epidural analgesia [17], one comparing SAPB with Erector Spinae Plane Block [18], and the last comparing SAPB with intravenous morphine [19]. In addition, we retrieved one prospective observational study [20], two retrospective studies [21,22], and two case series/reports [23,24].

Overall, we collected data from 197 patients treated with SAPB, at least 84 being critically ill. Of these, 28 (33%) patients received a single bolus of local anesthetic, while 56 (67%) were treated with continuous infusion. The bilateral approach was described in only three patients.

What emerged from our review was that, while information regarding analgesic protocols is frequently lacking, available data suggests very heterogenous management in terms of type, dose, and modality (bolus vs. continuous infusion) of local anesthetics, use of adjuvants, or association with other systemic analgesic therapies. In addition, relevant clinical details and outcomes (e.g., airway management, successful weaning, adverse effects) are often not reported.

Apart from the discussed limitations, the literature review suggests that SAPB provides significant pain relief, which might be comparable to epidural analgesia. Of note, only two papers discuss the benefit of this approach in promoting rapid weaning from mechanical ventilation [22,24], and the bilateral approach has been reported only in a few publications [22,23,24].

Importantly, only limited side effects of the SAPB were reported: one episode of hematoma [22], one infection [22], one catheter section [23], and five episodes of block failure [17,22]. Finally, one interruption of local anesthetic administration has been described in a patient with cardiac arrest to exclude a possible interfering factor [17].

Notably, these events are not specifically related to the SAPB and could occur whenever a perineural/perifascial or epidural catheter is positioned.

## 4. Discussion

We describe the case of a critically ill patient with severe thoracic pain from multiple rib fractures managed successfully with a continuous bilateral Serratus Anterior Plane Block. Furthermore, we present the results of a literature review concerning the SAPB in critically ill patients with traumatic rib fractures. Given its safety and ease of application, this technique may serve as a viable alternative to epidural analgesia in such cases.

Effective pain management is fundamental in patients with multiple rib fractures, particularly those who are critically ill. Pain can severely impair the ability to cough effectively, thereby increasing the risk of pneumonia. Additionally, it is vital to avoid the side effects of systemic opioids, such as sedation or central respiratory depression. We initially considered epidural analgesia to facilitate weaning from mechanical ventilation and to assess the patient’s neurological status without confounding factors. Although epidural analgesia is the gold standard, catheter placement can be challenging and poses risks such as falls, accidental extubation, and removal of drainage tubes or vascular catheters. Moreover, the risk of intraspinal hematoma formation after the administration of neuraxial anesthesia and analgesia is increased in patients who have received anticoagulant therapy or have a coagulation disorder. Other risk factors for the development of epidural or spinal hematoma include technical difficulty and multiple attempts. For these reasons, fascial blocks might be safer in this population of patients [25]. In our case, after a failed attempt by an experienced physician, we sought an alternative strategy due to the patient’s high requirements of opioids and sedatives.

The choice of a serratus plane block was supported by recent literature suggesting its non-inferiority to epidural or paravertebral blocks in chest trauma [20]. This finding may be due to the challenges in managing epidural catheters in critically ill patients, where pain assessment is complex and the dermatomeric spread of local anesthetic in the epidural space is difficult to evaluate. Furthermore, the hemodynamic instability often seen in these patients may prompt physicians to prescribe lower doses of local anesthetics. On the contrary, the serratus plane block is straightforward to perform, does not cause hemodynamic impairment, and allows for consistent dosing of local anesthetics, ensuring proper drug distribution. Other techniques, such as the erector spinae or paravertebral block, were also considered. However, we opted for SAPB, as we were more familiar with this approach and it does not require patient repositioning. Of note, our patient’s pelvic fractures limited mobility, and managing two posterior catheters in this situation would have been challenging. Finally, while the literature is scarce, there are currently no studies that demonstrate a superiority of the erector spinae or paravertebral block as compared to the SAPB. In addition, the bilateral SAPB provides easy access to the catheters, facilitating clinical assessment in case of suspected displacement or infection.

While the safety of a single serratus plane catheter is well-documented, less is known about the bilateral approach. Our patient had bilateral rib fractures, significantly impairing his weaning from mechanical ventilation, and considering the prediction of moderate to severe chest pain for several days, the positioning of perifascial catheters was indicated. Furthermore, the chest tube positioned after the mini-thoracostomy also represented a painful stimulus. Since the epidural catheter placement was unsuccessful, we opted for a bilateral serratus plane block. Although concerns about systemic toxicity from local anesthetics with this approach exist, we observed no signs or symptoms of toxicity. While current guidelines on maximum local anesthetic dosages are based on bolus administration, recent data suggest that continuous infusion is safe, even over extended periods [26,27]. Nonetheless, the risk of systemic toxicity should not be overlooked, particularly in patients with liver disease, where lower levels of plasma α1-acid glycoprotein increase the free fraction of local anesthetics [26,27]. A programmed bolus regimen, as supported by recent evidence, might reduce local anesthetic requirements and provide better pain control than continuous infusion [28], likely because each bolus creates a new block, whereas continuous infusion may struggle to maintain adequate spread within the fascial plane. Although available data indicate a good safety profile, some aspects specific to critically ill patients require attention. Continuous invasive monitoring can help identify hemodynamic signs of systemic local anesthetic toxicity, but sedation may mask early neurological symptoms. In our case, better pain control allowed us to keep the patient awake, making neurological assessment more reliable.

Beyond systemic toxicity, pneumothorax is another possible complication due to inadvertent pleural puncture [29]. Finally, a thoracic hematoma following serratus plane catheter placement was recently reported in a non-critically ill, anticoagulated patient, requiring surgical intervention [30]. A similar event was also reported in a critically ill patient [22]; however, it is not clear whether the complication required intervention or not. Notably, a similar complication related to an epidural catheter could have had devastating consequences, especially in critically ill patients who cannot communicate the onset of neurological deficits.

In the same direction, recent literature seems to confirm that the SAPB could have a central role in this setting. These data, however, include a variety of case reports and series or retrospective studies in which a concordance between therapeutic strategies does not emerge.

Despite differences in the therapeutic approach (choice of local anesthetic, dose, modality of infusion, use of adjuvants), the SAPB successfully controlled pain in critically ill patients with chest trauma. However, the abovementioned limits and the preliminary nature of most cited papers do not allow the drawing of conclusions. Indeed, clinical trials investigating the role of SAPB in critically ill patients with chest trauma to favor weaning from mechanical ventilation are highly warranted.

Our manuscript has some limitations that need to be mentioned. First, we did not perform an objective pain assessment using validated scales immediately before and after the bilateral block. Second, our search was limited to PubMed and Embase, which may have led to the omission of some relevant studies.

## 5. Conclusions

This case report underlines the importance of providing an analgesic alternative to our critically ill patients whenever the conventional approaches are not applicable since uncontrolled pain may worsen outcomes. Furthermore, while epidural analgesia is the first choice, it carries risks of failure and adverse events. Systemic opioids, though effective, can impair consciousness evaluation and delay weaning from mechanical ventilation, increasing intubation duration and the risk of complications. For our patient, the continuous bilateral serratus plane block within a multimodal analgesia plan proved to be an effective and safe strategy. Data from the literature suggest a possible central role of this safe and feasible technique. However, considering the available data, the possibility to draw consistent conclusions in critically ill, mechanically ventilated patients is limited.

## Figures and Tables

**Table 1:** Summary of available literature on the Serratus Anterior Plane Block (SAPB) for chest trauma in critically ill patients in the Intensive Care Unit (ICU).

Author	Study Design	Number of ICU Patients	Monolateral /Bilateral Approch	Number of Injured Ribs	Modality of Anesthetic Infusion	Drugs Used
T. Martinez (2019) [23]	Clinical cases	10	Both	3–22	Continuous infusion (seven patients) Single shot (three patients)	IB: 4 mg/kg lidocaine 1% (max 30 mL) CI: 0.15 mL/kg/h (max 12 mL/h) ropivacaine 0.2% SS: 0.4 mL/kg ropivacaine 0.325%
B. Riley (2020) [22]	Retrospective	3	Both	7 ± 4	Continuous infusion	Not specified
L. Beard (2020) [20]	Observational study	117 ^X^	Not specified	Not specified	Continuous infusion	Not specified
P. Bhalla (2021) [21]	Retrospective	14	Not specified	8 ± 3	Continuous infusion	IB: 25 mL ropivacaine 0.2% CI: 10 mL/h ropivacaine 0.2%
W. A. Abu-Elwafa (2021) * [19]	Randomized trial	20	Monolateral	Not specified	Single shot	SS: 15 mL bupivacaine 0.25% + 15 mL lidocaine 1%
D.Q. El Malla (2021) [18]	Randomized trial	25	Monolateral	5 [3; 6] ^^^	Single shot	SS: 19 mL bupivacaine 0.25% + 1 mL dexamethasone (4 mg/mL)
C. D’errico (2021) [24]	Case report	1	Bilateral	15	Continuous infusion	IB: 20 mL ropivacaine 0.375% CI: 6 mL/h ropivacaine 0.2%
A.R. Lundèn (2023) [17]	Randomized trial	7	Monolateral	5 ± 2	Continuous infusion	IB: 30 mL ropivacaine 0.2% for patients > 50 kg; 20 mL for patient < 50 kg CI: 0.1 mL/kg/h ropivacaine 0.2%

Acronyms: CI is continuous infusion; SS is single shot; IB is initial bolus; SD is standard deviation. ^X^ number of ICU not specified (24 intubated). ^^^ median and interquartile. * The study was conducted by the Anesthesia and Intensive Care Department; however, it is not clear if the SAPB was performed in patients admitted to the ICU.

## Data Availability

The data presented in this study are available on request from the corresponding author due to privacy. The raw data supporting the conclusions of this article will be made available by the authors on request.

## Supplementary Materials

The following supporting information can be downloaded at: https://www.mdpi.com/article/10.3390/jcm14061864/s1, Figure S1: Flow diagram.

## Abbreviations

The following abbreviations are used in this manuscript:

ICU	Intensive Care Unit
GCS	Glasgow Coma Scale
SAPB	Serratus Anterior Plane Block
DCO	Damage Control Orthopedics
CI	Continuous Infusion
SS	Single Shot
IB	Initial Bolus
SD	Standard Deviation
