# Supplementary material for: Bilateral Serratus Plane Block in a Critically Ill, Mechanically Ventilated Patient with Multiple Rib Fractures Due to Severe Thoracic Trauma: Case Report and Literature Review
Source: J Clin Med. 2025 Mar 10;14(6):1864. doi: 10.3390/jcm14061864 (PMC11943023; doi:10.3390/jcm14061864)
Supplement: Supplementary file 1 [file jcm-14-01864-s001.zip › jcm-3497624-supplementary.pdf]

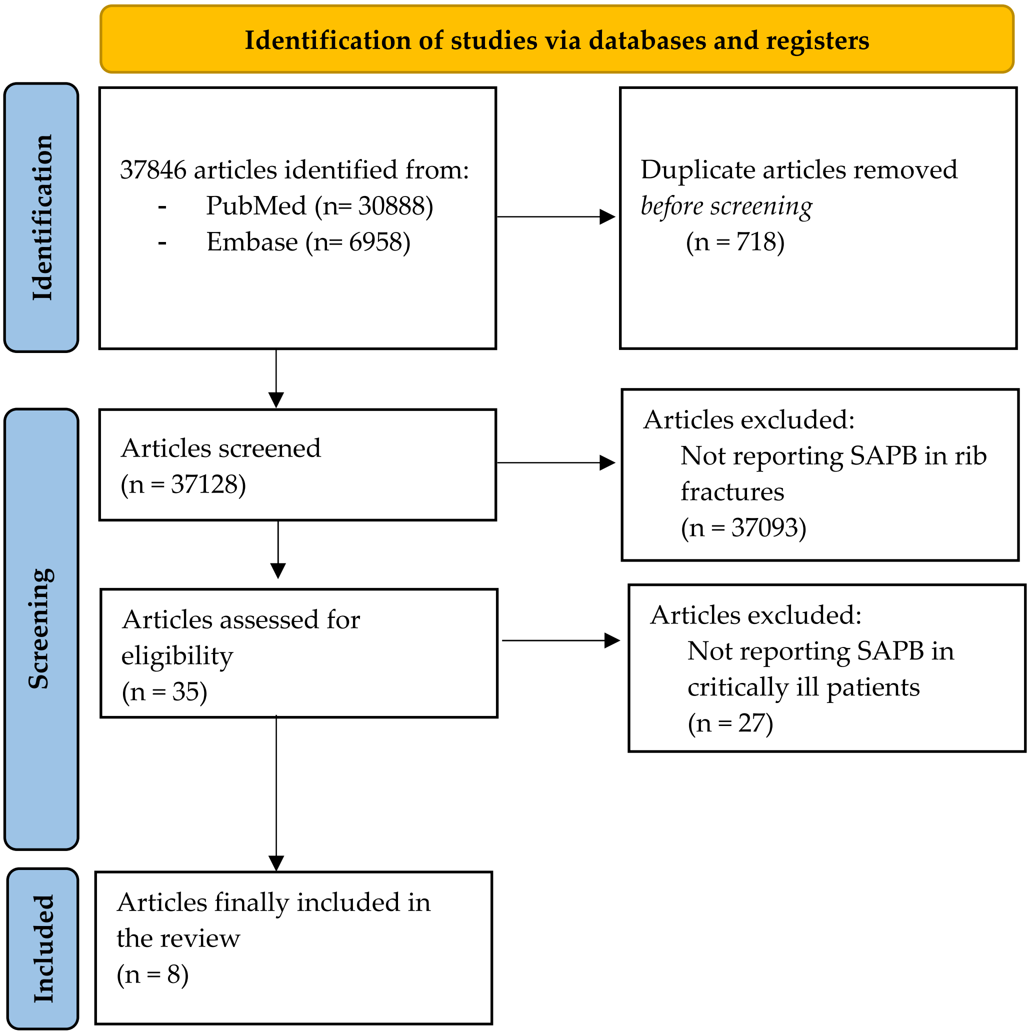

Figure S1. Flow diagram. Modified from the PRISMA 2020 guidelines [1], representing the screening process of the literature.

(1) PRISMA. *PRISMA 2020 flow diagram*. PRISMA. https://www.prisma-statement.org/prisma-2020-flow-diagram.
